# Implementing a user‐friendly format to analyze PRRSV next‐generation sequencing results and associating breeding herd production performance with number of PRRSV strains and recombination events

**DOI:** 10.1111/tbed.14560

**Published:** 2022-04-28

**Authors:** Giovani Trevisan, Michael Zeller, Ganwu Li, Jianqiang Zhang, Phillip Gauger, Daniel C.L. Linhares

**Affiliations:** ^1^ Veterinary Diagnostic and Production Animal Medicine Iowa State University Ames Iowa USA; ^2^ Programme in Emerging Infectious Diseases Duke‐NUS Medical School Singapore Singapore

**Keywords:** NGS, production outcomes, PRRSV, recombination

## Abstract

The open reading frames (ORF)5 represents approximately 4% of the porcine reproductive and respiratory syndrome virus (PRRSV)‐2 genome (whole‐PRRSV) and is often determined by the Sanger technique, which rarely detects >1 PRRSV strain if present in the sample. Next‐generation sequencing (NGS) may provide a more appropriate method of detecting multiple PRRSV strains in one sample. This work assessed the effect of PRRSV genetic variability and recombination events, using NGS, on the time‐to‐low prevalence (TTLP) and total losses in breeding herds (*n* 20) that detected a PRRSV outbreak and adopted measures to eliminate PRRSV. Serum, lung or live virus inoculation material collected within 3‐weeks of outbreak, and subsequently, processing fluids (PFs) were tested for PRRSV by RT‐qPCR and NGS. Recovered whole‐PRRSV or partial sequences were used to characterize within and between herd PRRSV genetic variability. Whole‐PRRSV was recovered in five out of six (83.3%) lung, 16 out of 22 (72.73%) serum and in five out of 95 (5.26%) PF. Whole‐PRRSV recovered from serum or lung were used as farm referent strains in 16 out of 20 (80%) farms. In four farms, only partial genome sequences were recovered and used as farm referent strains. At least two wild‐type PRRSV strains (wt‐PRRSV) were circulating simultaneously in 18 out of 20 (90%) and at least one vaccine‐like strain co‐circulating in eight out of 20 (40%) farms. PRRSV recombination events were detected in 12 farms (59%), been 10 out of 12 between wt‐PRRSV and two out of 12 between wt‐PRRSV and vaccine‐like strains. Farms having ≥3 strains had a 12‐week increase TTLP versus herds ≤2 strains detected. Farms with ≤2 strains (*n* 10) had 1837 and farms with no recombination events detected (*n* 8) had 1827 fewer piglet losses per 1000 sows versus farms with ≥3 PRRSV strains (*n* 8) or detected recombination (*n* 10), respectively. NGS outcomes and novel visualization methods provided more thorough insight into PRRSV dynamics, genetic variability, detection of multiple strains co‐circulating in breeding herds and helped establish practical guidelines for using PRRSV NGS outputs.

## 1 INTRODUCTION

Porcine reproductive and respiratory syndrome (PRRS) is a devastating swine disease caused by the PRRS virus (PRRSV) (Zimmerman et al., 2019). PRRS was initially described as ‘The mystery swine disease’, and since PRRSV isolation in the United States (U.S.) and in the Netherlands (Collins et al., 1992; Hill, 1990; Meulenberg et al., 1993; Terpstra et al., 1991; Wensvoort et al., 1991), PRRSV has spread globally (Brar et al., 2015; Murtaugh et al., 1995; Nelsen et al., 1999; Ramírez et al., 2019; Ramos et al., 2018; Rosendal et al., 2014; Trevisan et al., 2019b). The estimated annual production losses associated with PRRS were $664 million in the U.S. (Holtkamp et al., 2013) and $150 million in Canada (Mussel, 2011). In Europe, it was initially estimated to range between €3 and €160 per sow basis in a breeding herd (Nieuwenhuis et al., 2012) or between €75.72 and €650.09 per sow basis when considering the losses associated with all production phases (Nathues et al., 2017).

The PRRSV is an enveloped, single strained positive‐sense RNA virus in the order *Nidovirales*, family *Arteriviridae* (Kuhn et al., 2016). There are two distinct species of PRRSV: PRRSV‐1 (*Betaarterivirus suid 1*) and PRRSV‐2 (*Betaarterivirus suid 2*) (Kuhn et al., 2016; Zhang et al., 2019) distributed worldwide. The PRRSV‐1, colloquially known as the European type, has the Lelystad isolate (Meulenberg et al., 1993; Terpstra et al., 1991) as the prototype strain. The PRRSV‐2, colloquially known as the North American type, has the ATCC VR‐2332 (Collins et al., 1992) as the prototype strain. PRRSV‐1 and PRRSV‐2 share approximately 60% nucleotide identity (Allende et al., 1999; Kuhn et al., 2016; Zimmerman et al., 2019).

The whole‐PRRSV genome comprises ∼15.1 kilobases in length (Benfield et al., 1992; Collins et al., 1992; Kuhn et al., 2016; Meulenberg et al., 1993; Nelsen et al., 1999; Terpstra et al., 1991) and is organized into at least 10 open reading frames (ORFs) (Brar et al., 2015). The replicase region of the genome is encoded by the ORF1a and ORF1b, comprising about 75% of the genome, which is proteolytically processed into non‐structural proteins nsp1 to nsp12 (Dokland, 2010; Snijder & Meulenberg, 1998). The structural genome regions represent the other 25% of the genome and is organized into ORF2a, ORF2b, ORF3, ORF4, ORF5a, ORF5b, ORF6 and ORF7 genes (Dokland, 2010; Snijder & Meulenberg, 1998).

Within the whole‐PRRSV genome, the specific region ORF5b, also known and herein identified as ORF5, encodes the virus envelope protein and has been extensively used in molecular epidemiology and evolutionary studies to characterize distinct isolates (Brar et al., 2015; Lambert et al., 2019; Larochelle et al., 2003; Paploski et al., 2019; Paploski et al., 2021; Shi et al., 2010; Trevisan et al., 2021b; Wan et al., 2019b). The ORF5 gene comprises 603 nucleotide base pairs (bp) for PRRSV‐2 and 606 bp for PRRSV‐1, representing approximately 4% of the whole PRRSV genome and has been primarily used in the Americas to classify PRRSV‐2 strains according to restriction fragment length polymorphism (RFLP) patterns (Wesley et al., 1998) and genetic lineages (Lambert et al., 2019; Paploski et al., 2019; Paploski et al., 2021; Shi et al., 2010; Wang et al., 2019b), and to charactherize PRRSV strains in diversity studies (Alkhamis et al., 2016; Arruda et al., 2017; Brar et al., 2015; Ramos et al., 2018; Ramírez et al., 2019; Trevisan et al., 2021b). In U.S. veterinary diagnostic laboratories, sequencing of the PRRSV ORF5 region is performed using the Sanger technique (Sanger et al., 1977) which determines the consensus gene sequence in a sample and is not an efficient tool to sequence multiple viruses if more than one PRRSV strain is present (Harmon et al., 2019). In addition, evaluating only the ORF5 region misses the opportunity to better understand the whole‐PRRSV genome characteristics and perform additional investigations, for example, detection of recombination events (Lalonde et al., 2020). Next‐generation sequencing (NGS) is a technique used to sequence the whole‐PRRSV genome (Zhang et al., 2017) and has the potential to more thoroughly detect and characterize multiple PRRSV strains if present in the sample. Although NGS seems promising, the use and interpretation of NGS results by veterinary practitioners remains a dilemma (Risser et al., 2021).

The ability of more than one PRRSV‐2 strain or a PRRSV‐1 and a PRRSV‐2 strain to simultaneously infect a piglet has been demonstrated (Dee et al., 1997; Nilubol et al., 2014). Over time, detection of multiple, distinct PRRSV strains in a breed‐finish swine herd has been demonstrated by sequencing the PRRSV ORF5 genome region from individual serum and tissue samples (Dee et al., 2001). Anecdotal discussions between practitioners have commonly reported the perception of more than one PRRSV strain simultaneously circulating in a herd. To better support animal health decisions and interventions, there is a need to detect and characterize the presence of multiple PRRSV strains concurrently circulating in a herd and its association with key production performance outcomes.

Recently, processing fluids (PFs), a population sample type composed by the serosanguineous exudate recovered from testicles and tails during processing time, have been described (Lopez et al., 2018; Vilalta et al., 2018), and adopted as part of surveillance programs to detect PRRSV in newborn piglet populations and have also been validated to monitor breeding herds undergoing PRRSV elimination (Trevisan et al., 2019a). PF is currently one of the most used sample type for PRRSV monitoring in the U.S. (Trevisan et al., 2019b). By applying NGS to PFs, it would be expected to recover sequences in genomic regions in addition to ORF5 and detect one or more strains if present in the sample. This study evaluates the feasibility of applying NGS techniques in PF samples to investigate PRRSV genetic variability, detection of multiple, co‐circulating virus strains and recombination events and associate NGS results with production outcomes.

## 2 MATERIALS AND METHODS

### 2.1 Overview study design

This study was approved by the Iowa State University Institutional Animal Care and Use Committee under protocol number 19–118. This prospective field study was conducted in commercial breed‐to‐wean sow herds that faced a PRRSV outbreak and adopted measures to eliminate the PRRSV from the herd without depopulation. Upon study enrollment, participant herds were requested to share, when stored and available, samples that tested positive for PRRSV RNA by reverse transcription real‐time polymerase chain reaction (RT‐qPCR). Serum, lung tissue samples or live virus inoculation (LVI) material generated from lung or serum, collected within 3 weeks from the PRRSV outbreak were submitted for NGS to recover a farm‐specific PRRSV strain. After enrollment, participants agreed to prospectively collect PF for >90% of all processed litters within a week on an ongoing basis until the herd achieved low PRRSV prevalence (LP), defined as eight consecutive weeks with negative RT‐qPCR results for PRRSV. PF collected immediately after the outbreak, between 8 and 12 weeks, and the last samples with RT‐qPCR cycle threshold (Ct) < 30 before reaching LP were submitted to NGS. Recovered near complete‐PRRSV genomes at the outbreak were designated the farm referent strain and used for within and between herd PRRSV genetic comparisons and to investigate recombination events. Recovered near complete‐PRRSV genomes or contigs, that is, genome fragments detected in PF samples over time, were used for comparisons with the farm referent strain. The number of weeks required for the farm to achieve time to LP (TTLP) was associated with the number of detected strains and the presence of recombination events. Additionally, participant herds agreed to share key production indicators to calculate time to baseline production (TTBP) and total losses (TLs) measured as the total number of pigs not weaned per 1000 sows.

### 2.2 Sample collection and PCR testing

**FIGURE 1:**
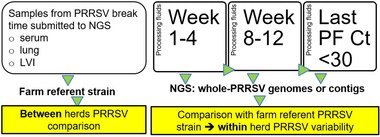
Timeline for sample collection, next‐generation sequencing (NGS) submission and expected NGS result comparisons. LVI, live virus inoculation material; PRRSV, porcine reproductive and respiratory syndrome virus; Ct, cycle threshold

*Sampling scheme*: When available, serum, lung or LVI material from the time of the outbreak with RT‐qPCR Ct < 30 were submitted for PRRSV NGS. After the outbreak, PF samples were collected by day of processing, stored frozen at −20°C at the farm, shipped to the Iowa State University Veterinary Diagnostic Laboratory (ISU‐VDL), pooled and tested for PRRSV‐1 and PRRSV‐2 by RT‐qPCR at the ISU VDL by week, on a fee for service basis and using commercially available RT‐qPCR kits. Based on time post‐PRRSV outbreak and RT‐qPCR Ct values, six PF samples per breeding herd were selected and submitted for NGS as follows: (a) two samples recently after the outbreak (week 1–4); (b) two samples around ten weeks after the outbreak (week 8–12); (c) post week 12, all PF samples were tested for PRRSV and stored at −80°C at the ISU‐VDL. After the farm achieved low prevalence, the last two PF samples with a Ct < 30 were retrospectively selected and submitted for PRRSV NGS (Figure 1).

### 2.3 NGS procedures

Indigenous samples were submitted to NGS. No viral isolation was performed to avoid the potential selection of a PRRSV strain. Software tools and procedures used for NGS were run with default parameters unless otherwise specified. Total nucleic acid extraction was performed using the MagMAX pathogen RNA/DNA kit and a KingFisher system (Thermo Fisher Scientific, MA) (Zhang et al., 2017). Double‐stranded cDNA was synthesized using the NEXTflex rapid transcriptome sequencing (RNA‐seq) kit (Bioo Scientific Corp., TX). Nextera XT DNA library preparation kit (Illumina, CA) with dual indexing was used to prepare the sequencing library. Fluorometer Qubit 2.0 (Life Technologies, Carlsbad, CA) was used to determine the concentration of the sequencing library that was then normalized to the concentration of 2 nM. The normalized library was sequenced on the MiSeq platform (Illumina) with a 2 × 250‐cycle MiSeq Reagent Kit V2 (Illumina). Sequencing reads were preprocessed using Trimmomatic v0.36 to remove adapters and trim low‐quality ends, depleting sequences with lengths of less than 36 nt, and sequencing quality analysis with FastQC (Andrews, 2010). Kraken v0.10.5‐β (Wood & Salzberg, 2014) was used to classify cleaned reads. Reads belonged to PRRSV were de novo assembled with SPAdes (v 3.5.0) as described previously (Wang et al., 2019a; Wang et al., 2019b).

### 2.4 Between herd PRRSV genetic comparisons

Recovered near complete‐PRRSV sequences representing strains within three weeks from the outbreak from serum, lung or LVI material were used to construct a phylogenetic tree for between participant farms comparison. Sequences were uploaded in Geneious Prime 202.0.1 (https://www.geneious.com), aligned using available MAFFT v7.450 default settings (Katoh, Misawa, Kuma & Miyata, 2002; Katoh & Standley, 2013), and a Neighbor‐Joining tree was built using the Tamura‐Nei genetic distance model in Geneious Tree Builder using default settings. The phylogenetic tree also included US commercially available PRRS‐modified live vaccine sequences for comparison with farm referent strains.

Additionally, structural and non‐structural genes (ORF1–7) and non‐structural proteins (nsp1–12) were annotated from the ATTC VR 2332 strain (GenBank U87392.3). ORF gene and nsp protein sequences were extracted and pairwise aligned using the MAFFT v7.450 available in Geneious, exported in a FASTA file format, and imported into R (R. Development Core Team, 2020) to extract and plot pairwise distances between different groups, ORF's and nsp's, using the Adegenet package (Jombart, 2008)

### 2.5 Recombination analysis

The near complete‐PRRSV genome recovered from serum, lung or LVI material at the time of outbreaks were used to investigate recombination events. Sequences from PRRSV‐2 MLV vaccines commercially available in the US and complete or near‐complete PRRSV‐2 genome sequences available in GenBank (https://www.ncbi.nlm.nih.gov/genbank) and identified from the US were downloaded in a FASTA file format. Downloaded sequences and farm recovered near complete‐PRRSV genome were collated into a single database and aligned using MAFFT v7.450 default settings (Katoh et al., 2002; Katoh & Standley, 2013). Recombination events and breakpoints were detected using the RDP4 software (Martin et al., 2015) and were supported by at least six of the seven available default parameters: RDP (Martin & Rybicki, 2000), GENECONV (Padidam et al., 1999), BootScan (Martin et al., 2005), MaxChi (Smith, 1992), Chimaera (Posada & Crandall, 2001), SiScan (Gibbs et al., 2000) and 3SEquation (Boni et al., 2007). The near‐complete PRRSV sequences with detected recombinations events were further plotted to confirm and visualize recombinant breakpoints using the SimPlot program (Lole et al., 1999). Due to the usage of indigenous samples for NGS, near complete‐PRRSV genome or contigs recovered from PF samples, a population sample type, were not evaluated for recombination events.

### 2.6 Detection of multiple PRRSV strains and within‐herd comparisons

Two distinct approaches were used for the detection of multiple PRRSV strains: (a) the default was used when the near complete‐PRRSV farm referent strain was recovered; (b) the alternative was used when no whole‐PRRSV sequence was recovered at the time of the outbreak.

*Using a whole‐PRRSV farm referent strain*: Contigs or whole‐PRRSV sequences recovered from PF samples were compared with the whole‐PRRSV farm referent strain using the NCBI nucleotide blast tool (https://blast.ncbi.nlm.nih.gov/Blast.cgi). The farm‐specific whole‐PRRSV genome referent sequence was entered as the query sequence and PF NGS outputs, that is, whole‐PRRSV or contigs as the subject sequences for a Blastn search. The hit tables for sequences producing significant alignments were downloaded in a file containing comma‐separated values. Vaccine‐like contigs were identified by repeating these procedures using the US commercially available PRRS modified live vaccines (MLV) strains, that is, Ingelvac PRRS^®^ MLV (GenBank AF066183), Ingelvac PRRS^®^ ATP (GenBank DQ988080), Prime Pac^®^ PRRS (GenBank DQ779791), Fostera^®^ PRRS (GenBank AF494042) and Prevacent^®^ PRRS (GenBank KU131568) as the query sequence.

Contigs were considered farm referent strain or a vaccine‐like if the similarity with the query sequence was ≥99%, potentially farm referent strain when had similarity ≥98 and <99%, and classified as wild‐type when similarity was <98% with the query sequence. Only contigs having a size equal to the smallest known PRRSV genome region, that is, nsp6 region, which is comprised of 48 bp were used in the analysis. The aligned positions of the contig sequences and their similarity with query sequence were plotted in a colour‐coded format using a Microsoft Excel spreadsheet.

*No whole‐PRRSV farm referent strain available*: When no whole‐PRRSV genome farm referent strain was available, the contigs recovered from PF at different sampling points were initially compared with the ATCC VR‐2332 strain, the PRRSV‐2 prototype strain (Collins et al., 1992), to determine genome positions. The contigs with the most extensive contiguous nucleotide genome coverage from distinct genome regions, recovered in a sample or from distinct samples, were then selected and re‐entered as query sequences on the NCBI tool to be compared with contigs recovered from other sampling events and covering the same genome region as the farm referent contig.

The presence of a second wild‐type PRRSV strain was considered when at least one of the two criteria was satisfied:

- The recovered contigs covered the entire length of at least one of the 5`UTR, nsp1‐5, nsp7, nsp9, ORF2a, ORF3, ORF4, ORF5, ORF6, ORF7 or 3`UTR genome regions and had <98% similarity with the farm referent strain. Genome regions were determined by annotating contigs from the ATCC VR‐2332 strain in Geneious.
- Contigs recovered from different genome regions and having a length equal to or greater than the smallest described functional PRRVS genome structure, that is, nsp6 comprised of 48 bp with <98% similarity with the farm referent strain recovered at different sampling points covering similar or distinct genome regions.

### 2.7 Association with production performance

The total number of strains detected in a herd and identification of recombination events were respectively evaluated for the association with TTLP, TTBP and TLs.

*TTLP* across groups was compared using Kaplan–Meier survival analysis (Kaplan–Meier) available on PROC Lifetest procedure in SAS 9.4 (SAS Institute Inc., Cary, NC). It was measured as the time in weeks it took for the farms to achieve low prevalence after the outbreak. Farms that dropped the study due to depopulation before 10 weeks post PRRSV outbreak were excluded from the analysis and, after 10 weeks, were censored. Between group, survival differences were determined by using a log‐rank test at a .05 significance level.

*TTBP* was measured as the time in weeks it took for the farms to recover the levels of pigs weaned per week as prior to the outbreak (Linhares et al., 2014). Exponential weighted moving average (EWMA) available in the PROC MACONTROL procedure from SAS 9.4 (SAS Institute, Inc., Cary, NC) was used to scan the database for changes from a baseline level. EWMA parameters were set to 3 sigmas and lambda of 0.4 (Linhares et al., 2014). The number of sigmas adjusts the width of upper and lower control limits, and lambda adjusts the assigned weight to the most recent variable value included in the data (Montgomery, 2009). Baselines were constructed based on the 26‐weeks previous to the initial PRRS outbreak to cover one complete breeding and two weaning cycles. The number of pigs weaned were considered outside of monitored parameters or returned to baseline if they were outside or returned to monitoring baseline parameters for at least two consecutive weeks (Linhares et al., 2014). TTBP across different groups was compared using the Kaplan–Meier survival estimates by using a log‐rank test at a .05 significance level.

TLs were calculated as the total number of pigs not weaned per 1000 sows from the PRRSV outbreak to when the herd returned to TTBP. TL was calculated as the cumulative sum of the number of pigs not weaned below baseline levels prior to the outbreak. TLs across different groups were compared using a generalized linear mixed model available in PROC GLIMMIX procedure in SAS using recombination and number of strains as explanatory variables and TLs as the dependent variable at a .2 significance level.

During the investigation period, no farm exclusion occurred since farms enrolled in this study did not experience outbreaks with other infections pathogen, for example, porcine epidemic diarrhoea virus.

## 3 RESULTS

This study enrolled breeding herds (*n* = 20) that experienced a PRRSV outbreak and adopted measures to eliminate the virus from the herd. Herds were located in five US states and were linked to eight production systems or veterinary clinics. Breeding herds had a median of 3065 productive sows (25th percentile 2475 and 75th percentile 3290). RT‐qPCR or NGS detected only PRRSV‐2.

**FIGURE 2:**
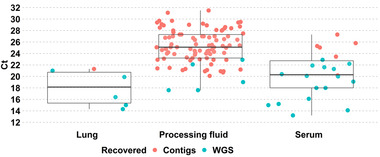
Success of recovering whole‐PRRSV genome sequences (WGS) or contigs (genome fragments) based on viral load. Ct values: (a) lung: median 18.2, 25th percentile 15.4, and 75th percentile 20.7; (b) processing fluids: median 25.1, 25th percentile 23.2, and 75th percentile 27.4; (c) serum: median 20.3, 25th percentile 18, and 75th percentile 22.7

**FIGURE 3:**
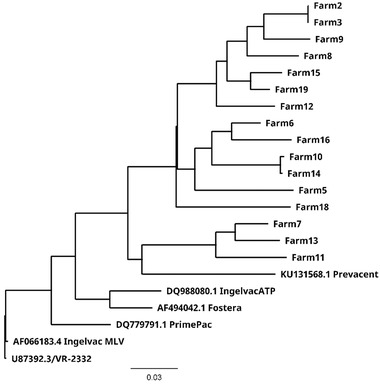
Neighbor‐Joining phylogenetic tree representing 16 farms where a whole‐PRRSV referent strain sequence was recovered. Strains for the commercially available PRRSV modified live vaccines in the United States and the PRRSV‐2 prototype strain ATCC VR‐2332 are also displayed. Tree was rooted by the ATCC VR‐2332 strain

A near complete‐PRRSV genome was recovered in five out of six (83.3%) lung samples, 16 out of 22 (72.73%) serum samples and in five out of 95 (5.26%) PF samples (Figure 2). A farm referent PRRSV strain was recovered from serum or lung in 16 out of 20 (80%) farms (Figure 3).

**FIGURE 4:**
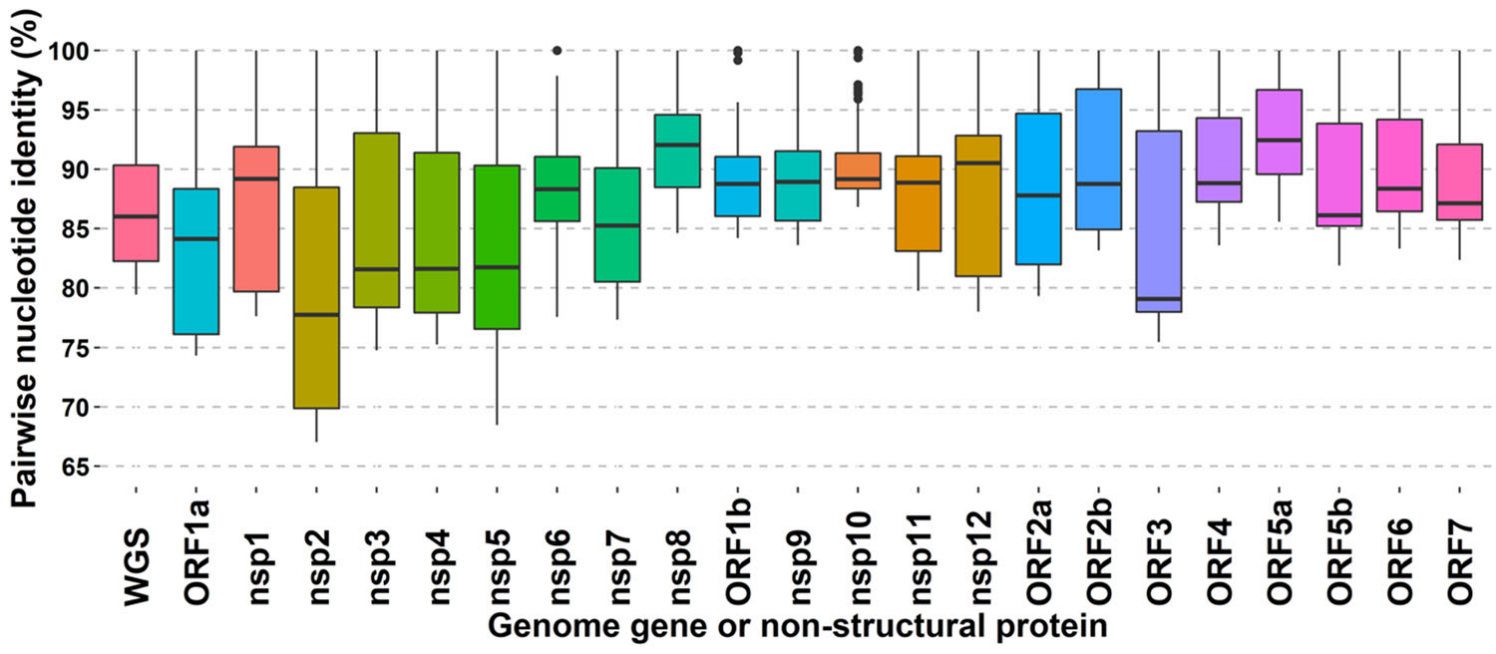
Pairwise comparison for farm PRRSV referent strains. Overall, whole‐PRRSV genome nucleotide identity is represented by the bar designated WGS. Non‐structural proteins nsp1–nsp8 are subregions of the ORF1a gene. The nsp8 is a subregion within the nsp9 codified in the ORF1a gene. Non‐structural proteins nsp9 (except nsp8 subregion), nsp10–nsp12 are ORF1b gene subregions. The ORF2b gene is an ORF2a gene subregion. ORF5b gene is also known as ORF5. The ORF5a is an ORF5 gene subregion

The sequences of 16 farm PRRSV referent strains were analyzed. The whole‐genome sequence nucleotide identity ranged from 79.4% (farm 11 and farm 16) to 100% (farm 2 and farm 3) with a median of 86%. Sequence analyses were further conducted at individual gene levels. The lowest nucleotide identity between herds was detected at ORF3 with 79.4% and non‐structural protein nsp2 (an ORF1a gene subregion) with 76.2% (Figure 4). The ORF5a, an ORF5 gene subregion, had the highest nucleotide identity at a median of 92.6%, followed by the non‐structural protein nsp8, another subregion of the ORF1a gene, at 92%. Additional statistics are presented in Table S1 for each gene and non‐structural proteins.

In addition to the whole‐genome sequences of 16 farm referent strains, a large contig (12,888 bp) recovered from PF sample of farm 1 was also included for investigation of recombination events. Recombination events between different PRRSV wild‐type strains were detected in 10 out of 17 (58.8%) farms. Recombination events involving two wild‐type strains and a PRRS MLV vaccine strain were detected in two out of 17 (11.8%) farms. Seven of 12 (58.3%) detected recombination events were encountered in farms having ≥3 PRRSV strains (Table 1).

**TABLE 1:** Number of PRRSV strains and recombination detection by farm

	Number of PRRSV strains and method of detection				Recombination type and referent strains	
	Wild‐type	Vaccine‐like	Total	Method	Type of recombination	Referent strains (GenBank|Isolatename)
Farm 1	2	1	3	Contigs	Wild‐type and wild‐type	KF724398.1|XW005 & MN073093.1|PRR21032‐S1‐L001
Farm 2	2	1	3	Whole‐PRRSV	Wild‐type and wild‐type	MF326996.1|IA/2015/ISU‐9 & MN073097.1|5606R‐S6‐L001
Farm 3	2	0	2	Whole‐PRRSV	Wild‐type and wild‐type	MF326996.1|IA/2015/ISU‐9 & MN073097.1|5606R‐S6‐L001
Farm 4, 17 and 20	2	0	2	Contigs	Not detected	
Farm 5	2	0	2	Whole‐PRRSV	Wild‐type and wild‐type	MN073111.1|PRR027983‐S27‐L001 & MF326990.1|NC/2014/ISU‐3
Farm 6, 11, 13 and 16	2	0	2	Whole‐PRRSV	Not detected	
Farm 7	2	1	3	Whole‐PRRSV	Not detected	
Farm 8	2	1	3	Whole‐PRRSV	Wild‐type and wild‐type	MF663706.1|IA14737‐2016 & FarmX
Farm 9	2	0	2	Whole‐PRRSV	Wild‐type and wild‐type	MF663706.1|IA14737‐2016 $ MN073180.1|7703R‐S6
Farm 10	1	0	1	Whole‐PRRSV	Wild‐type and vaccine	MN073097.1|5606R‐S6‐L001 & MF326996.1|IA/2015/ISU‐9 & KU131568.1 Prevacent
Farm 12	2	2	4	Whole‐PRRSV	Wild‐type and wild‐type	MF663706.1|IA14737‐2016 & MN073081.1|0752R‐S4
Farm 14	2	1	3	Whole‐PRRSV	Wild‐type and vaccine	MN073097.1|5606R‐S6‐L001 & MF326996.1|IA/2015/ISU‐9 & KU131568.1 Prevacent
Farm 15	1	0	1	Whole‐PRRSV	Wild‐type and wild‐type	MF663706.1|IA14737‐2016 & MF327001.1|IA/2015/ISU‐14
Farm 18	2	2	4	Whole‐PRRSV	Wild‐type and wild‐type)	KP283411.1|Minnesota4 & MN073097.1|5606R‐S6‐L001
Farm 19	2	2	4	Whole‐PRRSV	Wild‐type and wild‐type	MF663706.1|IA14737‐2016 & MF327001.1|IA/2015/ISU‐14

**FIGURE 5:**
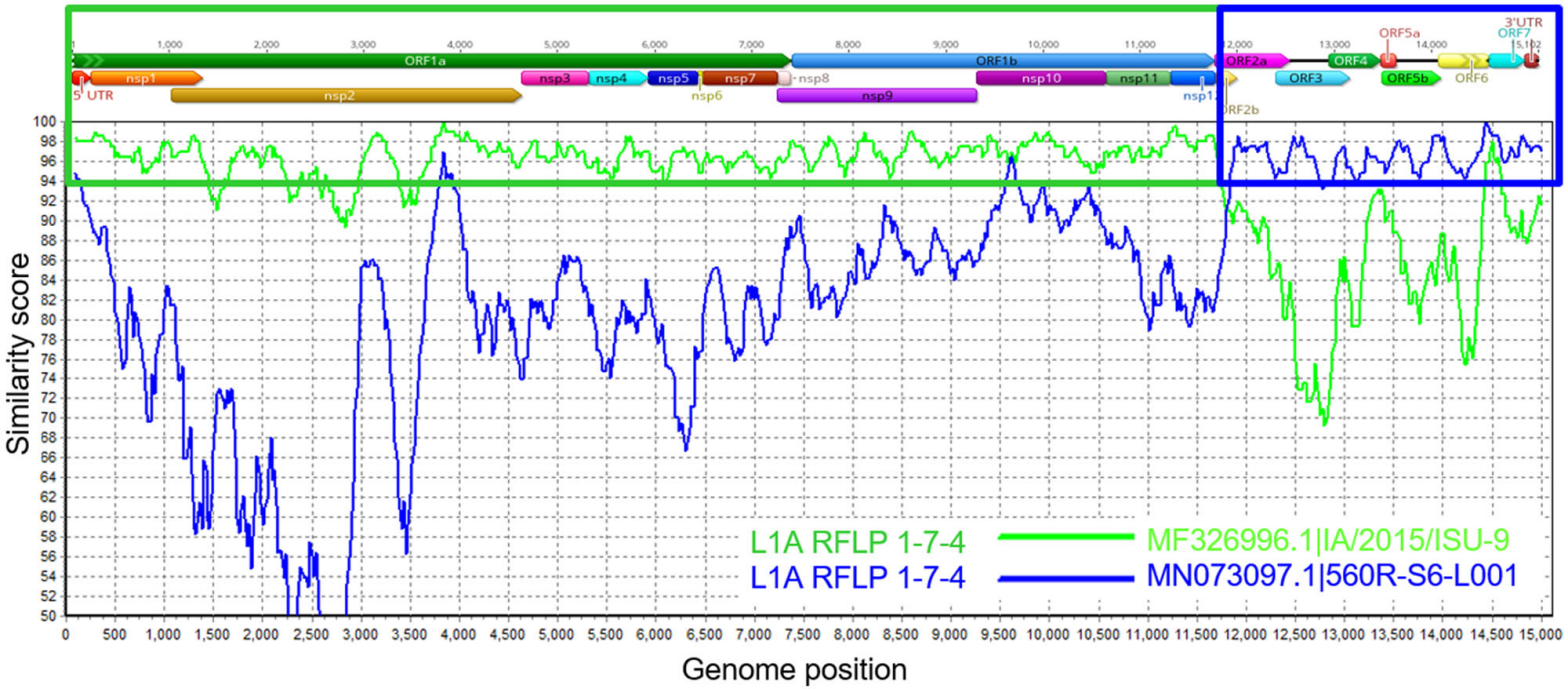
Similarity plot analysis using a farm referent strain as query sequence against two wild‐type Lineage 1A (L1A) PRRSV strains. PRRSV genome regions are presented at the top of the plot. Green and blue boxes outline the recombination breakpoints and genome regions that were derived from each of the parental and minor strains. Genome regions were plotted in Geneious and recombination events determined using SimPlot

**FIGURE 6:**
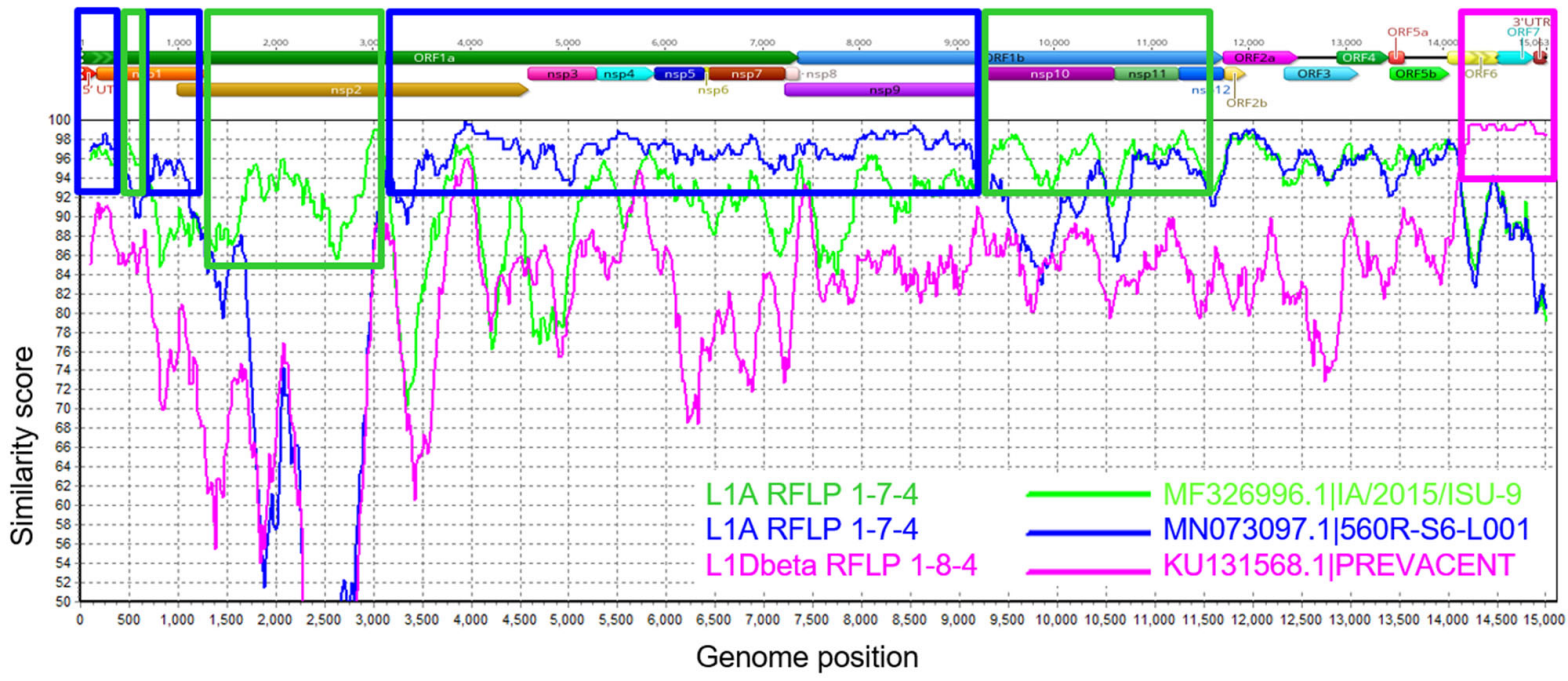
Similarity plot analysis using a farm referent strain as query sequence against two wild‐type Lineage 1A (L1A) PRRSV strains and Prevacent MLV vaccine strain. PRRSV genome regions are presented at the top of the plot. Green, blue and purple boxes outline the recombination breakpoint events and genome regions. Genome regions were plotted in Geneious and recombination events determined using SimPlot

Recombination breakpoints were identified at different genomic regions. As examples, Figure 5 showed a recombination format between two Lineage 1A RFLP 1‐7‐4 wild‐type strains, and Figure 6 presented a recombination involving two Lineage RFLP 1‐7‐4 wild‐type strains and a PRRS MLV vaccine strain. In Figure 5, the recombination breakpoint was at the end of the nsp12 protein. In Figure 6, distinct wild‐type recombination breakpoints were identified in the ORF1a and ORF1b genes, with an additional recombination event covering ORF6‐3′UTR genes involving the Prevacent vaccine strain.

The within‐herd PRRSV genetic comparison revealed the presence of at least two wild‐type PRRSV strains in 18 out of of 20 (90%) farms. Whole‐PRRSV genome recovered from serum or lung were used as farm referent strains in 16 out of 20 (80%) farms. For the other four farms, recovery of a near complete‐PRRSV sequence from serum or lung was unsuccessful either because there was no sample stored from the time of the outbreak or because the NGS procedure failed to recover a near complete‐PRRSV genome. Using the alternative approach of comparing contigs, it was possible to identify at least two wild‐type PRRSV strains simultaneously circulating in the herd. Contigs recovered from PF revealed the presence of PRRS MLV‐like strains in eight out of 20 (40%) farms. Five different cases representing different PRRSV outbreak scenarios and outcomes are described.

**FIGURE 7:**
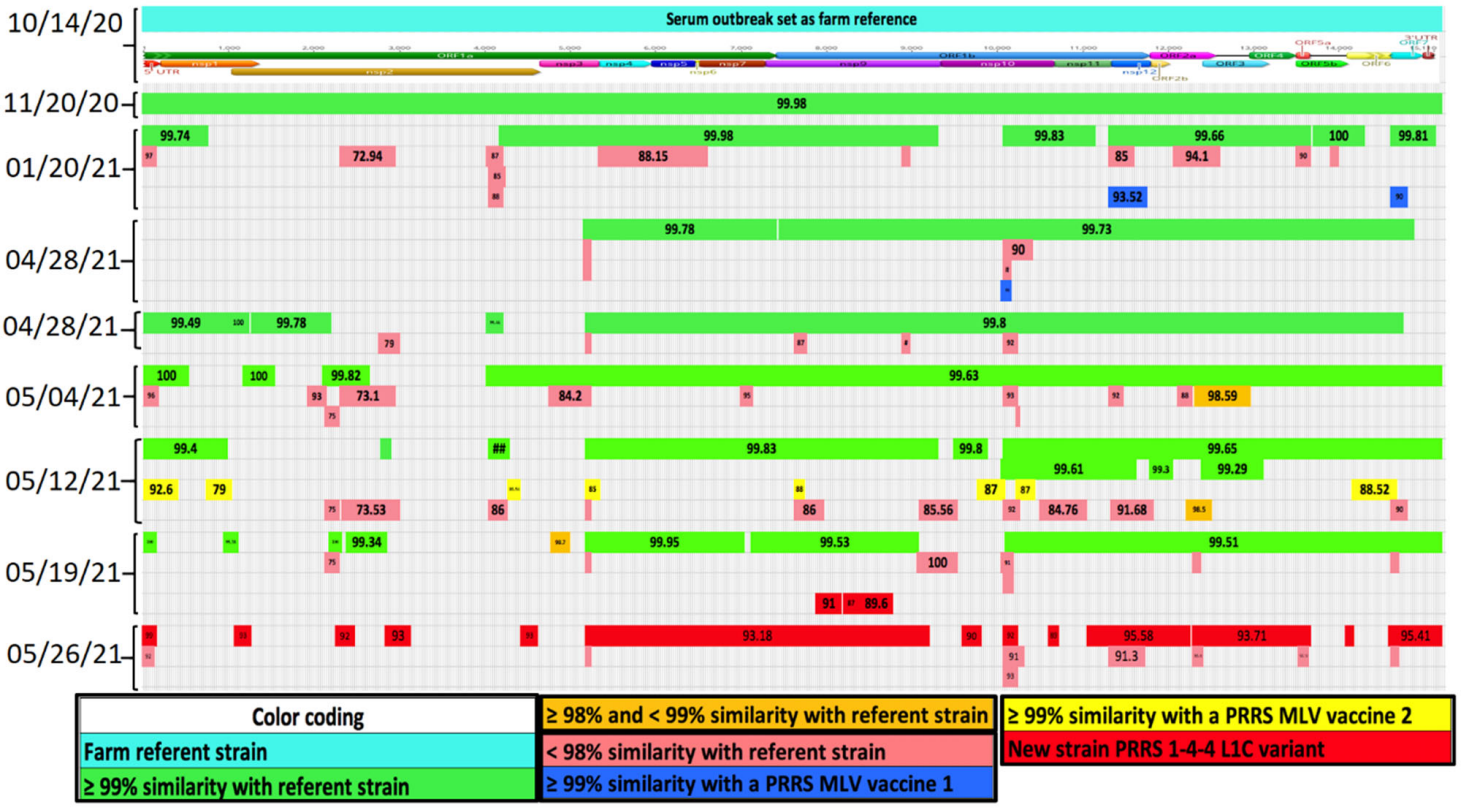
Comparison between a farm‐specific wild‐type PRRSV strain and genome fragments, that is, contigs, recovered from processing fluid samples characterizing the presence of at least five PRRSV strains. The structure at the top of the chart represents the farm referent strain position of open reading frames (ORFs) and non‐structural proteins (nsp). The percentage of nucleotide identity between contig and farm referent strain is labelled at the center of each contig. Colours represent the level of similarity with farm referent strain or with a commercially available PRRSV MLV vaccine. Contigs in green: >99%; in orange: ≥98 and <99%; in light red: <99% similarity with farm referent strain; in blue: >99% similarity Fostera; in yellow: >99% similarity with Ingelvac MLV; and in dark red: new PRRSV introduction

*Case A*: A 1700 breed‐to‐wean farm operating on a continuous breed‐farrow system, classified as Positive Stable (II‐A) for PRRSV according to the AASV scheme (Holtkamp et al., 2011), went through a PRRS rebreak with a Lineage 1A RFLP 1‐7‐4 PRRSV strain in October of 2020. Another introduction occurred in the same breed‐to‐wean farm in May 2021, with a strain sharing 99.4% with the PRRSV L1C variant strain (Kikuti et al., 2021; Trevisan et al., 2021a) and 92.8% nucleotide identity with the farm's referent strain. The farm had no reported PRRSV MLV use prior to or after the October 2020 outbreak. An LVI exposure with a PRRSV L1A strain occurred in April 2020, that is, 6 months prior to October 2020 outbreak. Replacement gilts were brought from an external multiplier source. A serum sample from October 2020 outbreak was used twice for LVI (L1A) of the whole herd at 2 and 5 weeks post‐outbreak. Herd closure took place 2 weeks after the outbreak. The farm‐referent PRRSV strain was recovered from serum, covering 15,101 bp and had evidence of recombination events detected between wild‐type PRRSV strains. A near complete‐PRRSV sequence covering 15,089 bp was also recovered on the first PF sampling point. In all subsequent sampling points, contigs from a second wild‐type strain representing different genome regions were repetitively recovered even after the introduction of the L1C variant strain in May 2021. On 20 January 2021, contigs from nsp12 and a fragment of the ORF7 gene and another contig from 28 April 2021, were Fostera‐like. On 12 May, eight contigs covering different genome regions were Ingelvac MLV‐like (Figure 7).

**FIGURE 8:**
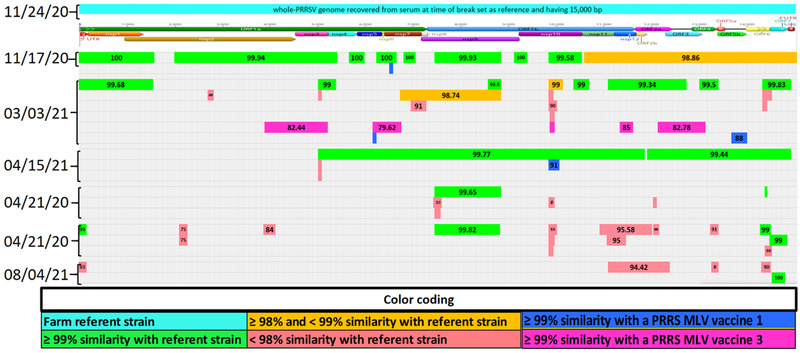
Comparison between a farm‐specific wild‐type PRRSV strain and genome fragments, that is, contigs, recovered from processing fluid sample characterizing the presence of at least 4 PRRSV strains. The structure at the top of the chart represents the farm referent strain position of open reading frames (ORFs) and proteins (nsp). The percentage of nucleotide identity between each contig and farm referent strain is labelled at the center of each contig. Colours represent the level of similarity with farm referent strain or with a commercially available PRRSV MLV vaccine. Contigs in green: >99%; in orange: ≥98 and <99%; in red: <99% similarity with farm referent strain; in blue: >99% similarity with Fostera; in purple: >99% similarity with Prevacent

*Case B*: A 3200 sow farm operating on a continuous breed‐farrow system, naïve for PRRSV, having the AASV classification Negative (IV) (Holtkamp et al., 2011), faced a PRRS outbreak with a Lineage 1A RFLP 1‐4‐3 PRRSV strain. At the time of the outbreak, the farm exposed sows and gilts to the PRRS Prevacent, and attenuated virus vaccine, and 4 weeks later with LVI (L1A strain). A serum sample from the time of outbreak was used for LVI and recovered a farm‐referent PRRSV strain covering 15,000 bp having evidence of recombination events detected between different wild‐type PRRSV strains. There was no history of MLV vaccine usage prior to the outbreak until the farm was administered the Prevacent MLV. Replacement gilts were internally produced and raised in an on‐site GDU. Herd closure took place four weeks after the outbreak. The first PF collected on November 17, 2020, (outbreak date) revealed a large region nsp11‐3′UTR with 98.86% nucleotide identity to the farm referent strain. A contig similar to the PRRS Fostera virus (another commercial vaccine based on attenuated PRRSV) was recovered in this sample and on 3 March and 15 April 2021, covering different genome regions. Contigs from additional wild‐type strains were consistently detected after March and were predominant when the farm reached low prevalence in the week of August 9 (Figure 8).

**FIGURE 9:**
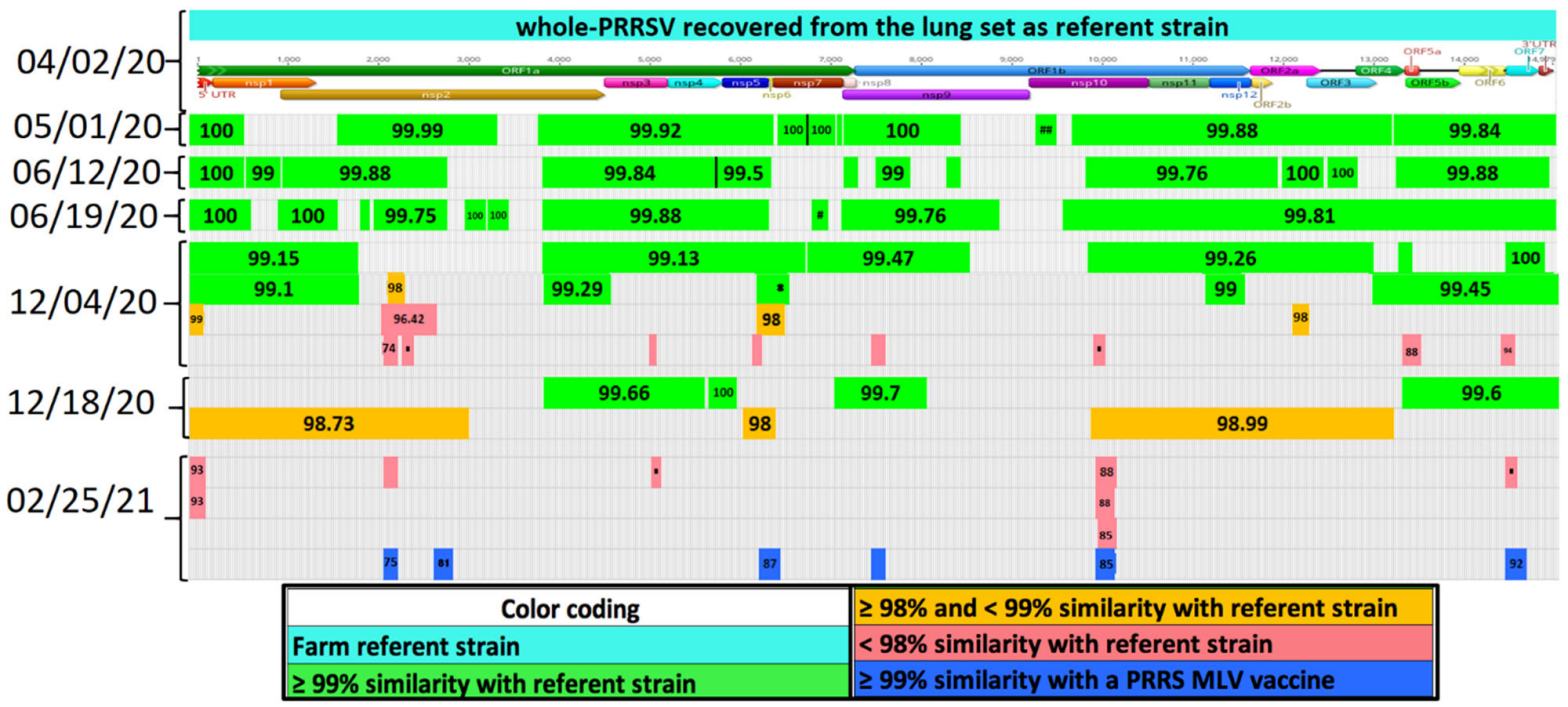
Comparison between a farm‐specific wild‐type referent PRRSV strain and genome fragments, that is, contigs, recovered from processing fluids samples characterizing the presence of at least three PRRSV strains. Farm referent strain genome positions open read frames (ORFs) and proteins (nsp) are presented at the top of the chart. The percentage of nucleotide identity between each contig and farm referent strain is labelled at the center of each contig. Colours represent the level of similarity with farm referent strain or with a commercially available PRRSV MLV vaccine. Contigs in green: >99%; in orange: ≥ 98 and <99%; in red: <98% similarity with farm referent strain; in blue: >99% similarity with Fostera

*Case C*: A 6000 sow farm operating on a continuous breed‐farrow system naïve for PRRSV, AASV classification Negative (IV) (Holtkamp et al., 2011), went through a PRRS outbreak with a Lineage 1A RFLP 1‐8‐4 strain in April 2020. No history of MLV usage was reported prior to or after the PRRSV outbreak. Replacement gilts were brought from an external source, and herd closure took place 7 weeks after the outbreak. A lung sample from outbreak time was used for LVI all breeding females and teaser boars 3 weeks after the outbreak. The farm‐referent strain recovered from lung covered 14,979 bp and had no evidence of recombination events. After the outbreak, the first three sampling points (1 May, 12 June and 19 June 2021) recovered farm referent strain contigs. In the week of 20 December 2020, a PRRSV ORF5 sequence recovered from serum collected from sows demonstrating abortions had 99% similarity with the strain detected at the time of the outbreak. On a sample from 18 December 2020, contigs ≥98 and <99% similarity with the farm referent strain were recovered. Preceding these events, the farm had 19 weeks with intermittent negative results and Ct > 30 on PF. On 4 December 2020, PF tested positive for PRRSV with a Ct of 27.3. Additionally, the EWMA monitoring (data not shown) revealed a spike in stillbirths and abortions since the end of November. The PF from 4 December was sent to NGS and recovered farm referent strain contigs, and contigs from the nsp2, nsp3, nsp5, nsp9, nsp10, the ORF5a and ORF6 genes that were neither farm referent nor vaccine‐like strains. These findings indicated the presence of a second wild‐type strain 3 weeks prior to the appearance of clinical signs, that is, abortions. Even though the farm had no history of PRRS MLV usage, the last sample with Ct < 30 in PF (25 February 2021) before the farm achieved low prevalence at week 54, recovered contigs from a second wild‐type and Fostera‐like strain (Figure 9).

**FIGURE 10:**
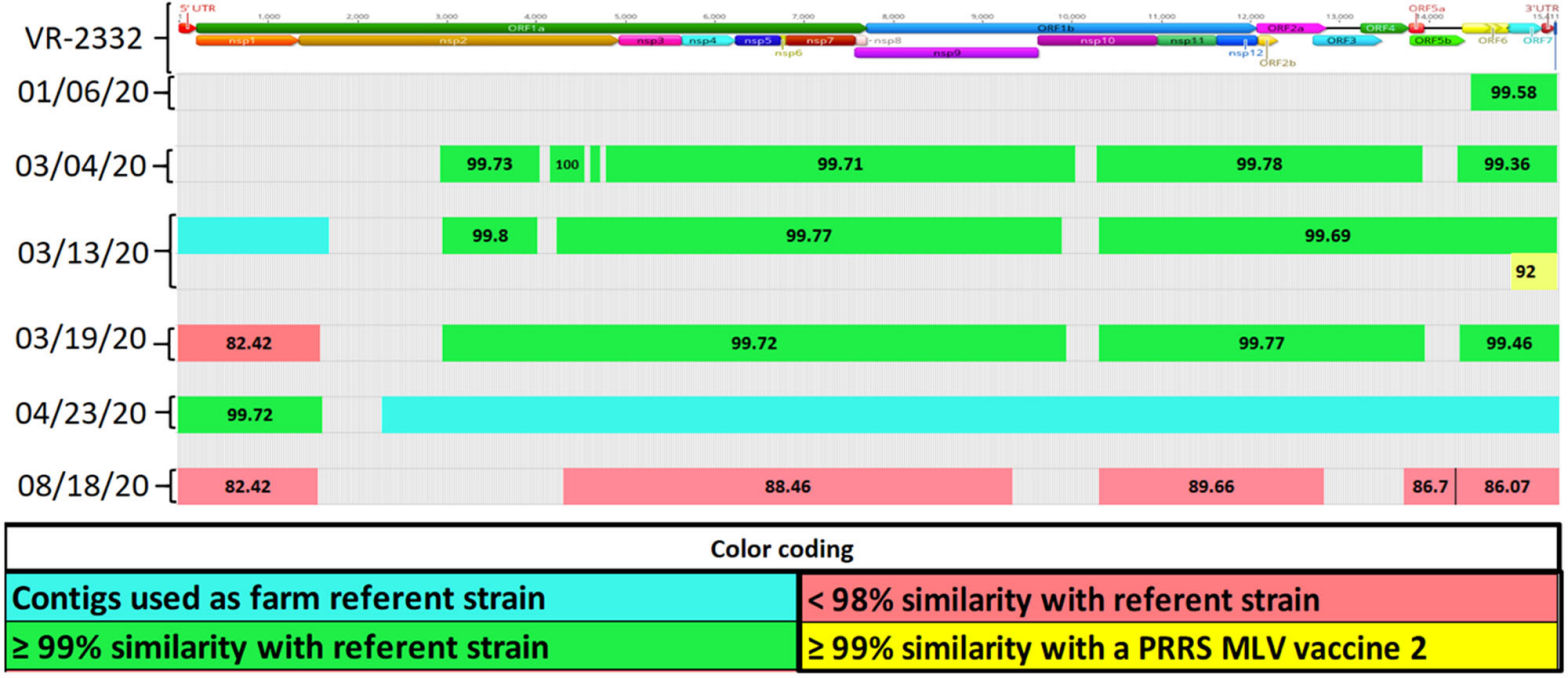
Comparison between a farm‐specific wild‐type PRRSV strain and genome fragments, that is, contigs, recovered from a processing fluid sample characterizing the presence of at least three PRRSV strains. The structure at the top of the chart represents the farm referent strain position of open reading frames (ORFs) and proteins (nsp). The percentage of nucleotide identity between each contig and farm referent strain is labelled at the center of each contig. Colours represent the level of similarity with farm referent strain or with a commercially available PRRSV MLV vaccine. Contigs in green: >99%; in orange: ≥98 and <99%; in red: <98% similarity with farm referent strain; in yellow: >99% similarity with Ingelvac MLV

*Case D*: A 3900 sow farm operating on a continuous breeding‐farrowing system, AASV classification Positive Unstable (I) (Holtkamp et al., 2011), faced a PRRS outbreak with a Lineage 1C RFLP 1‐2‐4 PRRSV strain. The farm adopted exposure to sows and gilts with Ingelvac ATP, an attenuated PRRSV vaccine, during the week of the outbreak. Replacement animals were brought from an external source and exposed to Ingelvac ATP twice. The herd was closed 1 week after the outbreak. No serum or lung samples were available from outbreak time for NGS. A PF sample from 19 March 2020, recovered one contig covering the 5′UTR and nsp1 genome regions. Another contig recovered from 23 April covered the nsp2‐3′UTR genome regions. Both contigs were set as farm referent strains. On 13 March 2020, a contig covering the ORF7 and 3′UTR region was Ingelvac MLV‐like. The entire farm was depopulated in September 2020. The last sample with PCR Ct value 29.5, that is, <30, was collected on 18 August and had recovered contigs from a second wild‐type strain. Even though the largest contig from 23 March 2020 had evidence of wild‐type strain recombination, this should be interpreted with caution since it was sequenced from a population sample, and the detected recombination could be an NGS artifact that could occur during sequence assembly of different genome regions for the two wild‐type strains circulating in the farm (Figure 10).

**FIGURE 11:**
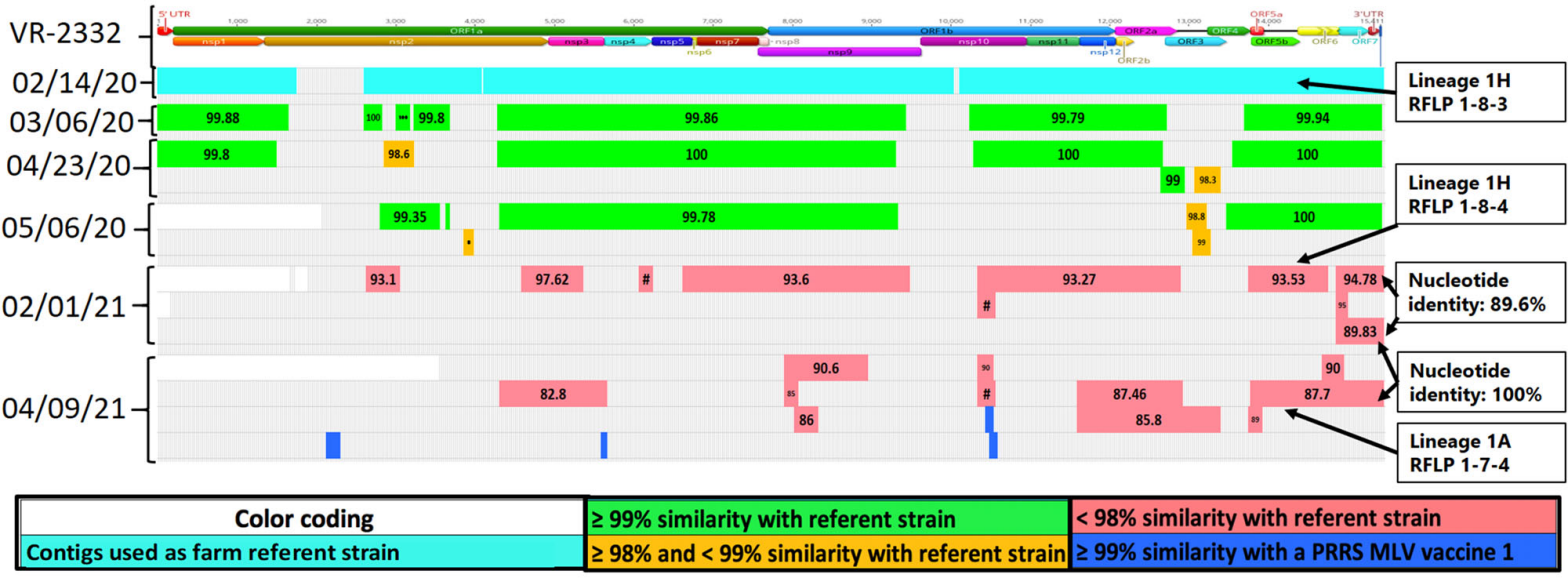
Comparison between a farm‐specific wild‐type PRRSV strains and genome fragments, that is, contigs, recovered from processing fluid samples characterizing the presence of at least three PRRSV strains. The structure at the top of the chart represents the farm referent strain position of open reading frames (ORFs) and proteins (nsp). The percentage of nucleotide identity between each contig and farm referent strain is labelled at the center of each contig. Colours represent the level of similarity with farm referent strain or with a commercially available PRRSV MLV vaccine. Contigs in green: >99%; in orange: ≥98 and <99%; in red: <98% similarity with farm referent strain; in blue: >99% similarity with Fostera

*Case E*: A 3000 sow farm operating on a bi‐weekly batch system, AASV classification Positive Stable (II‐A) (Holtkamp et al., 2011), went through a PRRS outbreak with a Lineage 1H RFLP 1‐8‐3 strain in January 2020. The herd had a history of Fostera PRRS vaccine usage. No serum or lung samples from outbreak time were available for NGS. Stored PF from 14 February 2020 recovered four large contigs that were included as farm referent strains. Samples from March, April and May recovered contigs with ≥98% nucleotide identity with farm referent contigs. Contigs with <99% nucleotide identity with referent contigs could indicate genetic evolution or the presence of other PRRSV strains. The farm did not reach low prevalence and had another outbreak with a Lineage 1H RFLP 1‐8‐4 PRRSV strain in January 2021. The sample from 1 February 2020 did not yield recovery of farm referent contigs. Two non‐farm referent contigs covering the ORF7 and 3′UTR region shared 89.6% nucleotide identity demonstrating the presence of another underlying PRRSV strain. On 9 April, a contig covering the entire ORF5‐3′UTR region had 100% match with one contig from 1 February. The ORF5 region from this contig was annotated, extracted and revealed a Lineage 1A RFLP 1‐7‐4 strain. There was no other evidence that this strain was circulating on the farm. Historically, a previous PRRSV outbreak had occurred in 2015 with a Lineage 1A RFLP 1‐7‐4 strain that shared 97.35% ORF5 nucleotide identity with the one recovered in 2021. Fostera PRRS vaccine‐like contigs were also recovered on 9 April (Figure 11).

Differences in production performance were encountered. Farms with ≤2 PRRSV strains (*n* = 11) reached stability 12 weeks before farms with ≥3 PRRSV strains (*n* = 8) (log‐rank 0.0459). Median TTLP and interquartile ranges (25th to 75th percentiles) for herds with ≤2 and ≥3 PRRSV strains were 30.8 weeks (27.0–37.0) and 42.8 weeks (26.0–54.0), respectively. No differences in TTLP were detected for farms with recombination events (*n* = 10) when compared with farms with no recombination (*n* = 8) events detected (log‐rank 0.7522).

No significant differences in TTBP were associated with the difference in the number of strains (log‐rank 0.5054) or detection of recombination events (log‐rank 0.4806). Farms with ≤2 PRRSV strains detected (*n* = 10), using the NGS technique, had 1837.4 fewer piglet losses per 1000 sows compared with farms with ≥3 PRRSV strains (*n* = 8). Farms with no recombination events detected (*n* = 8) had 1827 fewer piglet losses per 1000 sows than farms with recombination (*n *= 10) events detected Table 2.

**TABLE 2:** Total piglet losses based on the number of PRRSV strains and recombination events detected

Indicator		Total piglet losses per 1000 sows (95% confidence interval)	Difference	Tukey *p* value
Number of PRRSV strains	≤2	2432 (1697.6; 3166.7)	1837	.1154
	≥3	4269.6 (3448.3; 5090.9)		
Presence of recombination	No	2233.8 (1411.8; 3055.9)	1827	.1177
	Yes	4060.8 (3325.5; 4796.9)		

## 4 DISCUSSION

This study used 20 breeding herds that faced a PRRSV outbreak and adopted measures to eliminate the virus from the herd; those were followed over time and had PF samples submitted for PRRSV NGS. Results provided novel insights into farm‐level PRRSV detection, the occurrence of multiple strains circulating in a production system, and epidemiology dynamics. The relatively high viral concentration (low Cts) detected in serum and lung tissue samples may have contributed to the higher rate of near complete‐PRRSV genome sequencing success (∼15 kb) in these samples. Low viral concentration (high Cts), the potential presence of NGS inhibitors in the sample, and the presence of multiple similar PRRSV strains may have prevented the recovery of near complete‐PRRSV genomes in PF. When two genetically similar PRRSV‐2 (Zhang et al., 2017) or PRRSV‐1 (Vandenbussche et al., 2021) were present in the same sample submitted to NGS for whole‐genome sequencing, the recovery of contigs (i.e., genome fragments) of each virus strain was successful, and the consensus whole‐genome sequence was obtained; however, the recovery of a near complete‐PRRSV genome (∼15.1 kb) for each virus strain present in the sample has failed due to the short read length sequencing method and the inability to correctly align contigs with their respective viruses. Although this limitation occurred, it can be frequently based on the concentration of virus in the sample or sample type; the recovery of PRRSV genome fragments has provided valuable, scientific evidence to support the presence of multiple PRRSV strains simultaneously circulating in breeding herds.

The novel proposed approach of comparing contigs described in this report has provided valuable insights into PRRSV genetic diversity in breeding herds and the dynamic epidemiology that complicates the ability to control the virus effectively. Prior to using NGS, the presence of multiple PRRSV strains in herds could have gone undetected or remained unknown using traditional diagnostic methods and ORF5, that is, Sanger sequencing, which only provides the consensus sequence of the target gene.

Recently, the concept of applying NGS directly to field samples has been explored (Lalonde et al., 2020). There have been encouraging reports describing the potential recovery of a near complete‐PRRSV genome from field clinical serum samples with Ct up to 34.1, oral fluid with Ct up to 32.8 and tissue and lung samples with Ct up to 26.5 (Gagnon et al., 2021). RNA viruses, such as PRRSV, are challenging to sequence and characterize using high‐throughput sequencing technologies due to their genetic diversity, relatively short genome lengths and lack of conserved genome regions (Fitzpatrick et al., 2021). In this study, we have demonstrated the use of the Illumina MiSeq platform for high‐throughput sequencing output, and our results agreed with previous reports that near complete‐PRRSV genome sequences should not be expected for lung and serum samples with Ct > 25 (Zhang et al., 2017). Nevertheless, it is possible that using other commercially available high‐throughput sequencing platforms, long read‐length sequencing methods, and library sequence preparation kits may result in different levels of success of whole‐PRRSV genome recovery (Reuter et al., 2015).

Following the proposed criteria described in this report, we demonstrated the presence of multiple PRRSV strains simultaneously circulating in a breeding herd at different sampling points. These findings were consistent in different herds. NGS results are designed to provide the whole genome, not only the ORF5 region, and provide better insights into PRRSV genetic diversity, the number of strains circulating in breeding herds and its molecular characteristics. Sanger gene sequencing techniques (Sanger et al., 1977) are likely to be more successful in sequencing an entire gene, for example, ORF5 or ORF7; however, these traditional sequencing methods lack the ability to detect more than one sequence in a sample consistently but may represent a consensus sequence of the gene of interest (Harmon et al., 2019).

It has been previously reported that when more than one PRRSV strain is circulating in a population, the more aggressive strain with a higher replication rate will suppress the replication of the alternate virus, which ultimately represents the less predominant virus in a herd (Brar et al., 2015). The high replication rate varies due to a wide range of cell tropism (Zimmerman et al., 2019). Our study agrees with the literature, whereas the less predominant strains were detected more frequently when herds were repetitively sampled and approached low prevalence in repetitive circumstances.

Some herds were naïve for PRRSV (Holtkamp et al., 2011) detected a PRRSV outbreak and surprisingly had the detection of >1 wild‐type and vaccine‐like strains. Some herds did not have a history of attenuated PRRS MLV vaccine usage. It is possible that when a PRRSV outbreak occurs, there is the introduction of multiple PRRSV strains simultaneously, including vaccine strains. It is also possible that the introduction of new PRRSV strains may occur over time, and the initial strain has already stimulated the development of some levels of cross‐protective immunity suppressing the clinical expression of the newly introduced strain(s). Participant herds had a median of 3065 productive sows, which is a relatively large population. This could create an opportunity to support the circulation of different strains in subpopulations within the herd. Animals with increased age, like sows, are more resistant to PRRSV infection (Klinge et al., 2009). However, within a large population, individuals may still be able to replicate the virus and, in utero, transmit it to the progenies (Christianson et al., 1993; Nielsen et al., 2002; Terpstra et al., 1991). Adopting biomanagement and biocontainment procedures (Zimmerman et al., 2019), such as McREBEL (McCaw, 2000), should be encouraged to help minimize PRRSV transmission to other piglets and rooms, reducing potential recirculation of multiple strains on the farm, and perhaps lessen the PRRSV impact in the downstream flow. It is also possible that PRRSV outbreaks occurring in the downstream flow, attributed as lateral introductions, that is, from another pig farm, could potentially be leaking the less predominant PRRSV strains circulating in the breeding herd.

PRRSV recombination events, or mutations that occur due to the low fidelity of RNA virus replication, may lead to the emergence of new PRRSV strains (Yuan et al., 1999; Zimmerman et al., 2019). Here, we observed that herds with evidence of ≥3 PRRSV strains had most of the detected recombination events described in this study and may indicate a greater likelihood of recombination occurring in farms with ≥3 or more strains co‐circulating. Nevertheless, this work adopted the terminology vaccine‐like to describe contigs having similarity ≥99% to the commercially available MLV vaccines. As MLV vaccines are preceded by attenuating wild‐type strains, it is possible that some contigs could be part of a wild‐type strain. No contigs comparison with publicly available databased was made to rule this out.

More than half of enrolled herds had a recombination event detected that involved multiple wild‐type strains, including different genome regions. Due to the restricted number of complete or near complete‐PRRSV genomes available in GenBank, there is a potential to miss detection of recombination events between wild‐type strains. Alternatively, PRRSV MLV vaccine strains are well characterized, and the identification of recombination events between wild‐type and vaccine strains are more likely to occur (Risser et al., 2021; Wang et al., 2019a). In this study, we identified two farms that have been affected by a recombinant PRRSV that involved a wild‐type and vaccine strain. No outbreak investigation was conducted to discern the potential connection between those farms. NGS complete or near‐complete PRRSV genome outputs from individual indigenous lung and serum samples were used to investigate recombination events. Even though this study included one large contig obtained from processing a fluid sample in the recombination analysis, a prior plaque purification of PF sample could potentially help to isolate the virus and obtain a complete or near‐complete PRRSV genome. There is a potential for recombination events detected from a genome recovered from an indigenous population sample, for example, PF, oral fluid, to be an NGS artifact formed by distinct portions of two or more PRRSV strains if present in the sample.

NGS outputs have a competitive advantage when compared with ORF5 sequencing regarding the ability to better characterize PRRSV strains. For example, the strains presented in Figures 5 and 6 have evidence of being derived from similar ancestors, but in Figure 6, there was also recombination involving an MLV vaccine. Limited genetic comparison to one genome region may prevent the identification of relevant genetic characteristics of the strain. This study enrolled 20 breeding herds and revealed that multiple PRRSV circulating in a production system, that is, ≥3 PRRSV strains, was negatively associated with TTLP and TL and that recombinant strains were also negatively correlated with TTLP. It is possible that individuals cannot develop immunity for all the strains simultaneously, and subpopulations can maintain distinct PRRSV strains circulating on the farm and affecting animal performance.

Even though the use of NGS results to monitor PRRSV detection and genetic diversity is still evolving, a general recommendation involving this diagnostic technique would be to target the recovery of a farm‐specific whole‐PRRSV or near complete‐PRRSV genome at the time of the outbreak. Recovered strains can be used as farm referent strains for further comparison to help identify if new or genetically similar strains are circulating or were introduced into the herd, which helps populate a farm or production system‐specific library. For the initial NGS sequencing, samples that are more likely to yield higher viral concentrations, that is, low Ct values, for example, serum and lung, are preferred to help improve the success of acquiring the whole‐genome PRRSV sequence. Subsequent NGS can be applied to population samples like PF where further comparisons of recovered sequences, even if genome fragments, with the farm referent strain, is valuable and can help identify and characterize additional PRRSV strains circulating in a breeding herd.

## CONFLICT OF INTEREST

The authors declare no conflict of interest.

## Supporting information



Supplement Material

## Data Availability

Available data was provided in the manuscript; additional farm data is unavailable due to producer confidentiality.
