# Supplementary material for: Implementing a user‐friendly format to analyze PRRSV next‐generation sequencing results and associating breeding herd production performance with number of PRRSV strains and recombination events
Source: Transbound Emerg Dis. 2022 Apr 28;69(5):e2214–29. doi: 10.1111/tbed.14560 (PMC9790532; doi:10.1111/tbed.14560)
Supplement: Supplementary file 1 — Supplement Material [file TBED-69-e2214-s001.docx]

Supplemental table 1: Whole-genome, genes, and nos-structural proteins nucleotide pairwise comparison across farm referent strains (*n* = 16).

| Genomic region | Nucleotide identity | | | |
| --- | --- | --- | --- | --- |
|  | Median | Lowest | 25th percentile | 75th percentile |
| Complete genome | 86.0 | 79.4 | 82.2 | 90.3 |
| ORF1a * | 84.1 | 74.3 | 76.1 | 88.3 |
| nsp1 | 89.2 | 77.6 | 79.7 | 91.9 |
| nsp2 | 77.7 | 67.0 | 69.9 | 88.5 |
| nsp3 | 81.6 | 74.8 | 78.4 | 93.0 |
| nsp4 | 81.6 | 75.3 | 77.9 | 91.4 |
| nsp5 | 81.7 | 68.5 | 76.5 | 90.3 |
| nsp6 | 88.3 | 77.6 | 85.6 | 91.1 |
| nsp7 | 85.2 | 77.3 | 80.5 | 90.1 |
| nsp8** | 92.0 | 84.6 | 88.5 | 94.6 |
| ORF1b*** | 88.8 | 84.2 | 86.0 | 91.0 |
| nsp9 | 88.9 | 83.6 | 85.6 | 91.5 |
| nsp10 | 89.2 | 86.8 | 88.4 | 91.3 |
| nsp11 | 88.9 | 79.8 | 83.1 | 91.1 |
| nsp12 | 90.5 | 78.0 | 81.0 | 92.8 |
| ORF2a | 87.8 | 79.3 | 82.0 | 94.7 |
| ORF2b **** | 88.8 | 83.2 | 84.9 | 96.8 |
| ORF3 | 79.1 | 75.5 | 78.0 | 93.2 |
| ORF4 | 88.8 | 83.6 | 87.2 | 94.3 |
| ORF5b ***** | 92.4 | 85.6 | 89.6 | 96.7 |
| ORF5a | 86.1 | 81.9 | 85.2 | 93.9 |
| ORF6 | 88.4 | 83.3 | 86.4 | 94.2 |
| ORF7 | 87.1 | 82.3 | 85.7 | 92.1 |

* Non-structural proteins nsp1-nsp8 are an ORF1a gene subregions.

** The nsp8 is a subregion within the nsp9 codified in the ORF1a gene.

*** Non-structural proteins nsp9 (except nsp8 subregion), nsp10-nsp12 are an ORF1b gene subregions.

**** ORF2b gene is an ORF2a gene subregion.
***** ORF5b gene is also known as ORF5. The ORF5a is a ORF5 gene subregion.
